# Sustainable Healthy Working Life for All Ages—Work Environment, Age Management and Employability

**DOI:** 10.3390/ijerph20032712

**Published:** 2023-02-03

**Authors:** Kerstin Nilsson, Clas-Håkan Nygård, Tove Midtsundstad, Peter Lundqvist, Joanne Crawford

**Affiliations:** 1 Division of Occupational and Environmental Medicine, Lund University, 221 00 Lund, Sweden; 2 Division of Public Health, Kristianstad University, 291 88 Kristianstad, Sweden; 3 Department of Health Sciences, Faculty of Social Sciences, University of Tampere, 33014 Tampere, Finland; 4 Fafo Institute for Labour and Social Research, 0608 Oslo, Norway; 5 Department of People and Society, Swedish University of Agricultural Sciences, 230 53 Alnarp, Sweden; 6 School of Health, Victoria University of Wellington, Easterfield Building, Kelburn Parade, Wellington 6140, New Zealand

The proportion of elderly citizens is continuously increasing in most of the industrial world [1,2,3]. The current demographic trend is characterised by increased longevity and lower fertility rates, resulting in an increasingly ageing population. The retirement age in many countries is being postponed adapting the economic and budgetary implications of increased longevity to the new demographic distribution. Older people are encouraged to continue working and to participate in the labour force for as long as possible [1,2,3]. The demographic situation stresses the importance of factors that motivate older employees and self-employed individuals to keep working and maintain their employability until an older age, as well as encouraging the organisations and enterprises to care for their employees’ employability until an age older than the current retirement age [4,5,6,7].

There are a lot of factors that influence risks and problems, as well as employability and a healthy and sustainable working life for all ages at the individual organisational/enterprise and society level. The complexity of these factors has been identified in research. To make this complexity more manageable and comprehensible, the SwAge model has been used to organize these complex factors contributing to a healthy and sustainable working life for all ages in nine different areas of impact and determination. There are nine determinant areas identified in the SwAge-model [4,5], which are: (1) self-rated health, diagnoses, functional diversity; (2) physical work environment; (3) mental work environment; (4) work schedule, work pace, time for recuperation; (5) personal finances, work ability, employability; (6) personal social environment and work–life balance; (7) work social environment, discrimination, leadership and age management; (8) motivation, stimulation and satisfaction with work tasks; (9) knowledge, skills, and competence (Figure 1). This Special Issue will contribute to the development of our theoretical and practical knowledge in the domains that influence people’s working life.

This Special Issue aims to collect articles of high academic standard investigating the Sustainable Healthy Working Life for All Ages—Work Environment, Age Management and Employability. Numerous manuscripts were received, of which 13 papers passed the peer-review process and are presented here as reprints.

## Conclusion of the new knowledge

This Special Issue includes investigations related to a sustainable working life. 

A sustainable working life is hindered by the demographic development, which features more senior workers and the need to work until an older age. The aim of the qualitative study by Nilsson & Nilsson [8] was to investigate organisational measures and suggestions to promote and make improvements for a healthy and sustainable working life for all ages in an extended working life. Based on data from focus group interviews and individual interviews with 145 individuals, the study identifies several measures and actions that might increase employability: to promote a good physical and mental work environment; to promote personal financial and social security; to promote relations, social inclusion and social support in the work situation; and to promote creativity, knowledge development, and intrinsic work motivation. This concept is based on the spheres of determination in the theoretical SwAge-model (sustainable working life for all ages). The authors also present a tool for dialogue and discussion on the work situation and career development of the employee and argue that regular conversations, communication, and close dialogue are needed and are a prerequisite for good working conditions and a sustainable working environment, as well as to be able to manage employees and develop the organisation further. Managers’ attitudes to senior workers are important for a healthy and sustainable working life that lasts to an older age. A study by Nilsson & Nilsson [9] therefore evaluated work life factors that managers in the Swedish municipality sector believe are crucial for their employees working or wanting to work until age 65 or older. Based on cross-sectional data from 249 managers, the authors find that more managers believe employees can work (79%) than they believe want to work (58%) until age 65 or older. From the managers’ point of view, health, physical work environment, skills, and competence are the factors determining whether employees are able to work until age 65 or older, while insufficient social support at work and a lack of possibilities for relocations are factors that influence their willingness to work until age 65 or older. Hence, the authors concludes that supplementary strategies might be needed to contribute to employees being willing and able to participate in working life until an older age. Hovbrandt et al. [10] investigate the associations between different job types and social participation from a long-term perspective. Based on data from 1098 working respondents aged 55 at baseline and a 10-year follow-up when the respondents were retired, the analyses revealed that social participation varied by job type. Jobs with high decision latitude, as in active and relaxed jobs, seem to predict high social participation, even after cessation of employment. In addition, high social participation during working life is a predictor of high social participation from a long-term perspective, which also promotes healthy aging. Hence, a supportive work environment with possibilities for employees to participate in decision-making may support social participation both prior to as well as after retirement, and thus to healthy aging. A qualitative study by Sousa-Ribeirio et al. [11] conducted among older nurses (aged 55–65 years) aimed to study how they experienced their working life, especially their late career and retirement. The results showed that nurses planned to continue working until the age of 65 and beyond. When reflecting on their late-career decisions, nurses considered nine areas covering individual, work, and organizational factors as central to their ability and willingness to stay. Overall, the nurses had good health and were very satisfied and committed to their job and to the organization. They mentioned having both the professional and personal resources required to cope with the physical and mental job demands, which were perceived as motivational challenges, rather than hindrances. Jaldestadt et al. [12] examined retirement decisions among blue-collar workers in manufacturing across a multi-national company. Taking a systems-level approach, the study identified that at the macro level, national pension systems had an impact, as only one country allowed people to retire earlier and pensions were seen as tough. Factors that influenced early retirement decisions at the meso level were work organisation shift work; at the micro level, the primary concerns were physical work demands and psychosocial work environment and, at the individual level, workers’ health and the meaningfulness of their work. At all levels, attitudes towards older workers were crucial in either prolonging work or increasing the risk of retirement. Suggestions from this work include ensuring managers are trained in age management, enabling tailored work, and helping older workers prepare for retirement. 

The work environment in the education system is also important for a sustainable working life. School principals’ work situation was investigated by two studies. The objective of the study by Nilsson et al. [13] was to increase the knowledge regarding school principals’ work situations by examining the associations between various factors and the school principals’ assessments of their ability or desire to work until the age of 65 or older. The results showed that about 83% of the school principals stated that they could work and about 50% stated that they wanted to work until 65 years of age and beyond. Their exhaustion symptoms and experiences of an excessive burden, as well as their experiences of support from the executive management in the performance of their managerial duties, were of primary importance for whether the school principals wanted to work until 65 years of age and beyond. The study strengthens the robustness of the theoretical SwAge model regarding the investigated factors related to determinant factors for a sustainable working life and as a basis for developing practical tools for increased employability for people of older ages. Arvidsson et al. [14] investigated to what extent various work environment factors and signs of exhaustion were associated with reported intentions to change workplace among principals working in compulsory schools. The patterns of intended and actual changes in the workplace across two years were described, together with associated changes in occupational factors and signs of exhaustion. Supportive management was associated with an intention to stay, while demanding role conflicts and the feeling of being squeezed between management and co-workers (buffer function) were associated with the intention to change workplace. The principals who intended to change their workplace reported more signs of exhaustion. To increase retention among principals, systematic efforts are needed at the national, municipal, and local level, in order to improve their working conditions. A study by Schön Persson et al. [15] explored prerequisites for flourishing workplace relationships in a municipal healthcare setting for older people. As part of this process, they explored the staff’s suggestions as to how work relationships could be improved. Results showed that informal and formal meetings at work were shown to build positively perceived relationships. Suggestions for improving work relationships were also presented. This study contributes to workplace health promotion and has a salutogenic and participatory focus on how to explore workplace relationships as a resource. The flourishing concept shows how workplace relationships can be explored as prerequisites for workplace health promotion.

A sustainable working life was stressed by the effects of the COVID-19 pandemic on working life and the work environment. Nagel and Nilsson [16] used a questionnaire to investigate the association between, and the effect of, different factors in nurses’ work situations, organised based on the SwAge-model theories of a sustainable working life, associated with nurses’ work-related mental health diagnoses, before and during the COVID-19 pandemic. The results showed that lack of joy in the daily work, an increased workload, and lack of support from co-workers had an increased association with work-related mental health diagnoses. Kyrőnlahti et al. [17] examined the impact of home working on work ability in a sample of university workers during the COVID-19 pandemic. The study measured at three points after baseline measurements. The results identified that 75% of the sample had stable work ability; 18% of the sample had stable or improved work ability. Analysis identified that this improved work ability was associated with organisational support and significantly less reporting of work-related stress and musculoskeletal disorders. The final group of 8% of the sample had either poor or decreasing work ability. The analysis identified that decreasing work ability was associated with poor ergonomics at the home workplace, low levels of support from the organisation, high stress levels, and high levels of musculoskeletal pain. This highlights the factors that need to be implemented to support continued work ability for those required to work at home.

This Special Issue also includes systematic reviews and discussion papers regarding a sustainable working life. In their study, Ropponen et al. [18] updated information and explored definitions of “sustainable working life” via a systematic literature review and described working life trajectories based on the prevalence of sickness absence, disability pension, and unemployment in a Swedish twin cohort. They found 16 peer-reviewed articles published between 2007 and 2020. The most common definition of “sustainable working life” was the SwAge-model, which included a broad range of factors, e.g., health, physical/mental/psychosocial work environment, work motivation/satisfaction, and the family situation and leisure activities. The annual prevalence across years had a decreasing trend of unemployment over time stated Näsi et al., [19] whereas the prevalence of sickness absence had more variation, with a stable disability pension. They concluded that no consensus exists for a “sustainable working life,” meriting further studies. The paper by Deng et al. [20] proposes the development of new ways of measuring sustainable employability. This paper argues for the SwAge-model. By including environmental factors in the measurement of sustainable employability, you can then take into account digital exclusion, intrinsic work value, movement capital, and perceived employability; you can then develop and test measures in this framework. While these are developing concepts, future work can test these factors on the employed and the unemployed.

## Figures and Tables

**Figure 1:**
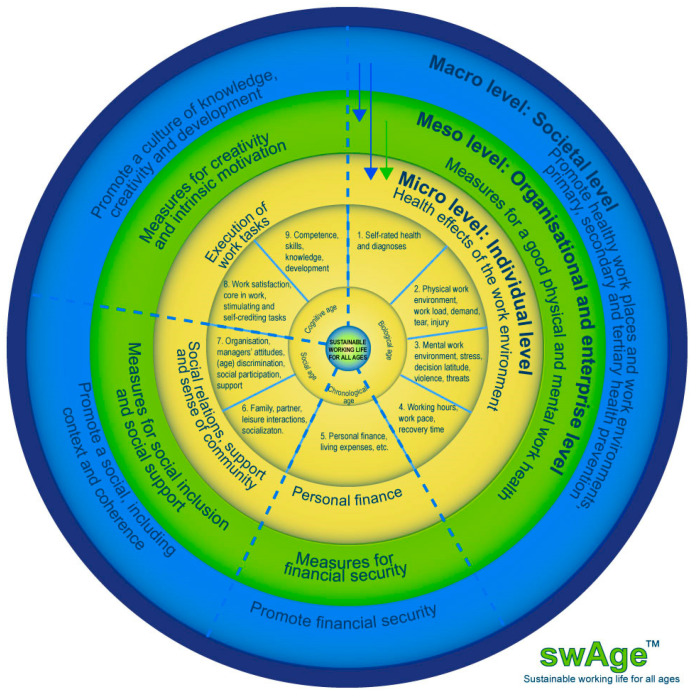
The SwAge-model.
